# Geopolitical Risk Evolution and Obstacle Factors of Countries along the Belt and Road and Its Types Classification

**DOI:** 10.3390/ijerph20021618

**Published:** 2023-01-16

**Authors:** Wei Hu, Yue Shan, Yun Deng, Ningning Fu, Jian Duan, Haining Jiang, Jianzhen Zhang

**Affiliations:** 1 College of Geography and Environmental Sciences, Zhejiang Normal University, Jinhua 321004, China; 2 Key Laboratory of Watershed Earth Surface Processes and Ecological Security, Zhejiang Normal University, Jinhua 321004, China; 3 Faculty of Geographical Science, Beijing Normal University, Beijing 100875, China

**Keywords:** the Belt and Road, geopolitical risk, entropy weight TOPSIS method, obstacle degree model, minimum variance method

## Abstract

As a great practice of building a community of shared future for mankind, the Belt and Road Initiative is facing geopolitical risk brought by great power games, regional conflicts and terrorism. It is an important mission of geopolitical research to scientifically deal with the geopolitical risk along the Belt and Road. This study systematically constructs the geopolitical risk assessment index system and analyzes the spatiotemporal evolution, obstacle factors and risk types of geopolitical risk of countries along the Belt and Road by using the entropy weight TOPSIS model, obstacle degree model and minimum variance method. The research results showed that: (1) From 2005 to 2020, the polarization of geopolitical risk in countries along the Belt and Road was very significant, and the overall trend of geopolitical risk tended to deteriorate. (2) The Middle East and Eastern Europe were the most important geopolitical risk zones along the Belt and Road, and Afghanistan, Iraq, Russia and Ukraine were the main high geopolitical risk centers, with significant risk spillover effects from these centers. (3) Terrorism and close relations with the United States were the most important obstacle factors for geopolitical risk in countries along the Belt and Road, and military intervention politics, trade dependence degree and foreign debt burden were important obstacle factors for geopolitical risk in countries along the Belt and Road. (4) Geopolitical risk along the Belt and Road can be divided into sovereign risk dominant type, sovereign and military risk dominant type, sovereign and major power intervention risk dominant type, and sovereign and military and major power intervention risk jointly dominated type, among which sovereign and military and major power intervention risk jointly dominated type was the most important geopolitical risk type. In order to scientifically deal with geopolitical risk in countries along the Belt and Road, it is necessary to strengthen geopolitical risk awareness, pay attention to the dominant geopolitical risk factors, strengthen the control of regional geopolitical risk spillover and formulate reasonable risk prevention and control scheme based on geopolitical risk types.

## 1. Introduction

As a great practice of building a community of shared future for mankind, the Belt and Road Initiative is creating a nonexclusive international network of regional economic cooperation [1]. However, not all countries believe that the Belt and Road Initiative is an economic cooperation framework. Some countries believe that the Belt and Road Initiative is a tool of China’s foreign policy, and the Belt and Road Initiative has strong geopolitical considerations [2]. The United States believes that the Belt and Road Initiative is China’s policy response to the “Asia-Pacific rebalancing strategy”, and that the Belt and Road Initiative will consolidate China’s position as a leading regional power [3]. India believes that the Belt and Road initiative is a long-term strategic Initiative, which is aimed at transforming China’s current economic strength into diplomatic influence [2]. Japan views the Belt and Road from the perspective of competing for its own global investment plan and is worried that the Belt and Road Initiative will dilute Japan’s economic and political influence along the Belt and Road [4]. In fact, in the process of reshaping the geopolitical environment around China, the Belt and Road Initiative has led to the active participation of various forces in the geopolitical game out of their own interests, resulting in the spillover of regional geopolitical risk [5]. The geopolitical risk brought by the terrorist attacks in the China–Pakistan Economic Corridor, the ethnic and religious conflicts in Afghanistan and the Tigray conflict in Ethiopia have seriously threatened and hindered the advancement of many Belt and Road Initiative construction projects. In view of the complex geopolitical risk situation along the Belt and Road, we must grasp the evolution law of geopolitical risk along the Belt and Road, clarify the key risk factors of geopolitical risk along the Belt and Road, avoid and predict geopolitical risk along the Belt and Road and promote the healthy and orderly development of the Belt and Road.

Geopolitical risk is a reflection of geopolitical shock. On the one hand, geopolitical risks are manifested in ethnic conflicts, religious conflicts, economic dependence, political turbulence and other impacts on national sovereign security [6,7]. On the other hand, geopolitical risks are presented as military interventions, armed threats, major power interventions, terrorism and other threats to national military security [8,9,10]. Therefore, geopolitical risk is the impact of strategic interactions among geopolitical bodies based on their geographical location, resource endowment, economic structure, military power and other elements such as cooperation, competition, confrontation and containment in a specific geographical area on national security with sovereign security and military security as the core. Domestic and international scholars have concentrated their research on geopolitical risks on issues such as the process of occurrence, spatial differentiation, driving mechanisms, impact effect and assessment tools [11]. In terms of the process of occurrence, the outbreak of geopolitical risk is a process in which external geopolitical factors and internal conflict factors are intertwined [12]. In terms of spatial differentiation, there are significant regional differences in the sensitivity of different regions to geopolitical risk [13]. In terms of driving mechanisms, major power intervention, external conflicts, terrorism, political turbulence, ethnic and religious conflicts, and economic dependence are all driving the evolution of geopolitical risk [14,15,16]. It is worth noting that nontraditional security factors such as economic development, social stability, climate change and information technology can also influence geopolitical risk by affecting national stability. However, national sovereign security and military security are always at the core of geopolitical risk [17,18,19,20]. In terms of the impact effect of geopolitical risk, the geopolitical risk will cause fluctuations in international finance, global energy and global food supply on the macro level [21,22], and will cause enterprises, investors and consumers to adjust their own business behavior, investment strategies and consumption structure on the micro level [23,24,25]. In terms of assessment tools, geopolitical risk assessment has gradually shifted from qualitative to quantitative and visual analysis [26,27,28]. Indicators such as the global risks awareness index, risk attention, political system index, economic quality index and models such as the gravity model, systems analysis model and nonlinear autoregression model have been used for geopolitical risk assessment [29,30]. Geopolitical risk radar maps and global geopolitical risk maps are more and more widely used in geopolitical risk visualization [31]. In addition, technological tools such as global news event tracking technology, remote sensing monitoring and geo-setting situational awareness are providing important support for geopolitical risk monitoring and prediction in the Middle East region, South China Sea region, Korean Peninsula and other global geopolitically sensitive regions [32].

The Belt and Road Initiative not only covers the geopolitical "broken zones" such as Central Asia and Southeast Asia, but also focal areas of great power competition such as the South China Sea, the Middle East and Eastern Europe [33,34]. It also covers Afghanistan, Syria, Myanmar, Sudan and other countries with high incidence of terrorism, ethnic and religious conflicts. These make the geopolitical risk situation along the Belt and Road extremely serious [35,36]. As a tool for geo-economic to influence geopolitics, the Belt and Road Initiative cannot avoid the geopolitical game behind it, and the geopolitical competition behind the Belt and Road Initiative is very likely to induce geopolitical risk [5]. From a country perspective, the distribution of political risk along the Belt and Road is highly concentrated, especially in Afghanistan, Iraq and Myanmar [37]. In terms of projects, the frustration of major Belt and Road projects such as the shelving of the Myitsone hydropower project in Myanmar, the suspension of the China–Malaysia railroad project and India’s interference with China’s construction of Jaffna wind and solar energy projects in Sri Lanka is mainly due to geopolitical risk rather than economic risk [12]. From the perspective of enterprises, geopolitical risk has hindered the process of “going out” of Chinese enterprises, especially the investment and cooperation of Chinese enterprises in countries along the Belt and Road countries [38]. From the perspective of scale politics, the Belt and Road Initiative is full of multi-scale games between different countries, and geopolitical risks in the reconstruction of the global scale and local scale cannot be ignored [39,40].

These studies have important guiding significance for the prevention and control of geopolitical risk in the Belt and Road. However, the majority of these studies are qualitative analyses of geopolitical risk dynamics and evaluations of single risk factors from the perspective of international politics or international relations [41]. There is a lack of quantitative assessments of geopolitical risk and classifications of geopolitical risk types from the perspective of geography. Therefore, this study addresses the following three questions: (1) How can we achieve a multidimensional and multifactor comprehensive assessment of geopolitical risk from the perspective of geography? (2) How can we identify the main factors of geopolitical risk along the Belt and Road? (3) How can we scientifically classify geopolitical risk types along the Belt and Road? Solving these problems is conducive to promoting geopolitical risk research from the perspective of geography and can also provide a decision-making reference for the prevention and control of the geopolitical risk of the Belt and Road Initiative. To solve these problems, this study constructs a multidimensional and multifactor geopolitical risk index system from the perspective of geography, analyzes the spatial and temporal evolution of geopolitical risk in countries along the Belt and Road using the entropy weight TOPSIS method, analyzes the main obstacle factors of geopolitical risk using the obstacle degree model and classifies geopolitical risk types along the Belt and Road using the minimum variance method.

This study is structured as follows. In Section 1, the research background of geopolitical risk along the Belt and Road and a review of relevant literature is introduced. In Section 2, the research method, research area and research data are introduced. In Section 3, the evolution of geopolitical risk in countries along the Belt and Road is analyzed. In Section 4, the geopolitical risk obstacle factors of the countries along the Belt and Road route are analyzed. In Section 5, the geopolitical risk types of the countries along the Belt and Road are classified. In the last section, the conclusions and policy implications are presented.

## 2. Research Methods and Data Sources

### 2.1. Indicator System

The existing geopolitical risk assessment is mainly carried out from political, economic and social perspectives [42]. The geopolitical risk assessment from the political perspective is mainly based on national governance indicators such as political stability, legalization level and corruption control [43]. The geopolitical risk assessment from the economic perspective is primarily based on trade dependence degree, investment dependence degree and energy dependence degree [44]. The geopolitical risk assessment from the social perspective is mainly based on terrorism, religious conflicts and ethnic conflicts [45]. However, the core of geopolitical risk is sovereign security risk and military security risk, and geopolitical risk assessment, whether from a political perspective, an economic perspective or a social perspective, should be based on the kernel of geopolitical risk and avoid obscuring the kernel of geopolitical risk with indirect influencing factors of geopolitical risk. Therefore, this study assesses geopolitical risk from sovereign security risk and military security risk. Since major power intervention brought about by geostrategic competition among great powers or the geopolitical game of regional great powers can threaten both national sovereign security and national military security, geopolitical risk assessments must consider the impact of major power interventions. Therefore, this study constructs a geopolitical risk assessment index system for countries along the Belt and Road from sovereign risk, military risk and major power intervention, and assigns positive and negative directions based on the role of indicators on geopolitical risk(Table 1).

At the sovereign risk level, political risk, economic risk, energy risk and ethnoreligious risk are the main factors that induce geopolitical risk from within the country. The greater the political stability, the lower the probability of geopolitical risk caused by political events or political conflicts, and this study chose political stability to portray political risk. In terms of the economic risk dimension, national economies are interdependent in economic globalization, and excessive investment dependence and trade dependence can easily trigger economic turmoil, threatening economic sovereign security. Therefore, this study selects investment dependence degree and foreign trade dependence degree that reflect the country’s foreign economic dependence to describe the country’s economic sovereignty risk. Trade dependence is expressed by foreign trade dependence degree, and investment dependence degree is expressed by the proportion of net inflows of foreign direct investment. In the dimension of energy risk, energy security emphasizes that the normal energy demand for national survival and development has a stable guarantee, which is an important support for national sovereign security. Energy dependence degree reflects the security situation of national energy sovereignty. In the ethnoreligious risk dimension, social unrest brought by religious conflicts and ethnic clashes will directly endanger national sovereignty and induce geopolitical risk. In this study, we chose ethnic conflict intensity and religious conflict intensity to portray the sovereignty risk in ethnic and religious aspects. At the level of military risk, military intervention in politics will directly subvert state power, and violent armed threats and terrorist activities directly undermine national defense security. This study reveals military risk using the intensity of military interventions in politics, the intensity of violent armed threats and the global terrorism index, which reflect military risk. At the level of major power intervention, external conflicts such as diplomatic sanctions, tariff barriers, external wars, alliance relations, foreign debt burden and aid dependence and other acts of major power interventions can cause sovereign security and military security changes and affect geopolitical risk. External conflict risk spillover will directly cause the spread of geopolitical risk, and this study portrays external conflict using an external conflict index that reflects external pressures such as tariff barriers, diplomatic sanctions, international humanitarian interventions and border disputes. As a global hegemonic power, the United States intervenes most in global and regional affairs, and the geopolitical risk of the countries along the Belt and Road cannot ignore the United States factor, so this study chooses the relationship with the United States allies to portray the extent of the United States intervention. The higher the foreign debt pressure and aid dependence, the higher the likelihood of geopolitical risk induced by major power intervention. This study uses the ratio of foreign debt stock and net official development assistance received to reflect the risk of major power intervention.

### 2.2. Research Methodology

#### 2.2.1. Entropy Weight TOPSIS Method

The entropy weight TOPSIS method is a combination of the traditional technique for order preference by similarity to ideal solution (TOPSIS) method and the entropy method. The TOPSIS method is used to evaluate the research object according to the relative closeness between the research object and the ideal solution [46]. The entropy weight TOPSIS method can not only assess the level of geopolitical risk by comprehensively measuring the distance between the actual level of the evaluation object and the ideal level, but also overcome the shortcomings of subjective assignments which are more subjective. Therefore, this study adopts the entropy weight TOPSIS method to measure the geopolitical risk of the countries along the Belt and Road. The specific calculation steps are as follows:

(1) Index standardization. In order to eliminate the limitation of indicator dimension, we use the range standardization method to nondimensionalize the indicators in different positive and negative directions and obtain the standardized matrix *x_ij_*:
  (1) $$x_{ij}=\frac{x_{ij}-x_{\min}}{x_{\max}-x_{\min}}\left(i=1,2,3,\ldots ,m,j=1,2,3,\ldots ,n\right)$$
  (2) $$x_{ij}=\frac{x_{\max}-x_{ij}}{x_{\max}-x_{\min}}\left(i=1,2,3,\ldots ,m,j=1,2,3,\ldots ,n\right)$$
  (3) $$X_{ij}=\left[\begin{array}{cccc} x_{11} & x_{12} & \cdots & x_{1n}\\ x_{21} & x_{22} & \cdots & x_{2n}\\ \cdots & \cdots & \cdots & \cdots \\ x_{m1} & x_{m2} & \cdots & x_{mn} \end{array}\right]$$
  where *x_ij_* is the standardized value, *i* represents the *i*-th country and *j* represents the *j*-th evaluation object. *i* and *j* are integers and 1 ≤ *i* ≤ 74, 1 ≤ *j* ≤ 13. *m* is the number of countries and *n* is the number of evaluation objects.
(2) Determine the index weights. Calculate the weight values among the index data using the entropy weight method:
  (4) $$W_{j}=\left(w_{1}w_{2}\cdots \left.w_{n}\right)\right.$$
(3) Calculate the optimal solution set and the inferior solution set:
  (5) $$X^{+}=\left\{x_{1}^{+},\right.x_{2}^{+},x_{3}^{+},\ldots ,\left.x_{n}^{+}\right\},x_{j}^{+}=maj\left\{x_{\mathrm{ij}}\right\}\;\left(i=1,2,3,\ldots ,\left.m\right)\right.$$
  (6) $$X^{-}=\left\{x_{1}^{-},\right.x_{2}^{-},x_{3}^{-},\ldots ,\left.x_{n}^{-}\right\},x_{j}^{-}=m\mathrm{in}\left\{x_{\mathrm{ij}}\right\}\;\left(i=1,2,3,\ldots ,\left.m\right)\right.$$
  where *X*^+^ is the optimal solution of the *j*-th evaluation object, indicating that the country geopolitical risk is at the highest value. *X*^−^ is the inferior solution of the *j*-th evaluation object, indicating that the country geopolitical risk is at the lowest value.
(4) Calculate the distance between the geopolitical risk of a single country and the optimal solution set and the inferior solution set:
  (7) $$D_{i}^{+}=\sqrt{\sum_{j=1}^{n}w_{j}{\left(x_{ij}-\left.x_{ij}^{+}\right)\right.}^{2}}\left(i=1,2,3,\ldots ,\left.m\right)\right.$$
  (8) $$D_{i}^{-}=\sqrt{\sum_{j=1}^{n}w_{j}{\left(x_{ij}-\left.x_{ij}^{-}\right)\right.}^{2}}\left(i=1,2,3,\ldots ,\left.m\right)\right.$$
  where *D_i_*^+^ is the closeness of the *i*-th country to the optimal solution. The smaller the *D_i_*^+^, the closer the geopolitical risk of country *i* is to the optimal solution, and the higher the geopolitical risk of country *i* is. *D_i_*^−^ is the closeness of the *i*-th country to the inferior solution. The smaller the *D_i_*^−^, the closer the geopolitical risk of country *i* is to the inferior solution, and the lower the geopolitical risk of country *i* is.
(5) Calculate the relative closeness of geopolitical risks in a single country:
  (9) $$C_{i}=\frac{D_{i}^{-}}{D_{i}^{+}+D_{i}^{-}}(i=1,2,3,\ldots ,m)$$
  where *C_i_* is the geopolitical risk level of the i-th country, 0 ≤ *C_i_* ≤ 1. The greater the *C_i_*, the closer the geopolitical risk of country *i* is to the optimal solution, and the higher the geopolitical risk of country *i*. In order to facilitate comparison, this study refers to relevant studies and classifies the geopolitical risks of countries along the Belt and Road into five levels [46]. When 0.00 < *C_i_* ≤ 0.192, it is a very low risk level; when 0.192 < *C_i_* ≤ 0.365, it is a low risk level; when 0.365 < *C_i_* ≤ 0.455, it is a medium risk level; when 0.455 < *C_i_* ≤ 0.652, it is a high risk level; when 0.652 < *C_i_* ≤ 1, it is a very high risk level.

#### 2.2.2. Obstacle Degree Model

Although the entropy weight TOPSIS method can assess the level of geopolitical risk, it cannot identify the key obstacle factors of geopolitical risk [47]. In order to further assess the key factors of geopolitical risk of each country, this study introduces the obstacle degree model into the analysis of the geopolitical risk of countries along the Belt and Road. By analyzing the obstacle degree of each geopolitical risk index, we can reveal the effect degree of each risk factor on the geopolitical risk of the countries along the Belt and Road. The specific calculation process is as follows:

(1) Calculate the gap between individual risk factors and the theoretical maximum value of the risk factor:
  (10) $$O_{ij}=1-x_{ij}$$
  where *O_ij_* is the gap between individual risk factors and the theoretical maximum value of the risk factor, and *x_ij_* is the standardized value of the risk indicator.
(2) Calculate the effect of individual risk factors on the geopolitical risk of each country:
  (11) $$I_{j}=\frac{O_{ij}\cdot w_{j}}{\sum_{j=1}^{n}O_{ij}\cdot w_{j}}$$
  where *I_j_* is the obstacle degree of risk factor *j* and *w_j_* is the weight of risk factor *j*. The larger the obstacle degree *I_j_*, the stronger the effect of risk factor *j* on geopolitical risk.

#### 2.2.3. Minimum Variance Method

Based on the contribution of the risk factors identified by the obstacle degree model, this study assesses the degree of contribution of each subsystem to geopolitical risk and then classifies geopolitical risk types with the minimum variance method. The minimum variance method uses variance to describe the dispersion degree of data around the average value and defines the closest value between the actual distribution and the theoretical distribution as the minimum variance [48]. The smaller the variance between the actual distribution and the theoretical distribution, the closer the actual distribution type is to the theoretical distribution type. The minimum variance method can effectively identify the gap between the actual distribution type of geopolitical risk and the theoretical distribution type and can also clarify the characteristics of geopolitical risk types. The specific classification process is as follows:

(1) Calculate the effect of each subsystem on geopolitical risk:
  (12) $$U_{z}=\sum I_{j}$$
  where *U_z_* is the obstacle degree of subsystem *z*, *z* is an integer and 1 ≤ *z* ≤ 3.
(2) Calculate the closeness between the geopolitical risk of each country and the theoretical distribution types of geopolitical risk:
  (13) $$S_{z}{}^{2}=\frac{1}{n}\sum_{z}^{n}\left(U_{z}-{\left.{\bar{U}}_{z}\right)}^{2}\right.$$
  where *S_z_*^2^ is the variance, *U_z_* is the effect of subsystem *z* on geopolitical risk and ${\bar{U}}_{z}$ is the effect of subsystem *z* on geopolitical risk in theoretical distribution types. As the geopolitical risk system in this study is composed of sovereign risk, military risk and major power intervention, theoretically, there are single system risks, double system risks and three system risks in this study. In theory, the risk value of one subsystem accounting for 100% of the total geopolitical risk is the “single system risks”, the risk value of two subsystems accounting for 50% of the total geopolitical risk is the “double system risks” and the risk value of three subsystems accounting for 33.3% of the total geopolitical risk is the “three system risks”. According to the minimum variance theory [48], compare the variance *S_z_*^2^ value of country *i* when ${\bar{U}}_{z}$ is 100%, 50% and 33.3%, and the geopolitical risk type of ${\bar{U}}_{z}$ corresponding to the minimum value of *S_z_*^2^ is the geopolitical risk type of country *i*.

### 2.3. Data Sources

**Figure 1:**
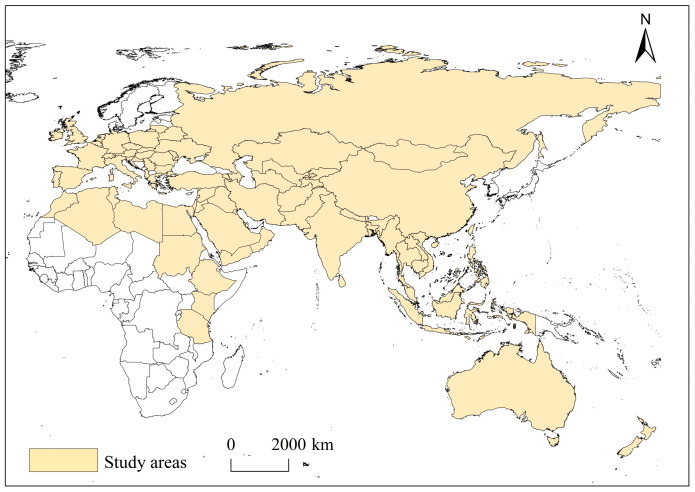
Study areas. Note: the map is drawn to the standard map of GS (2016) 1667 that come from the standard map service system of the ministry of natural resources of the people’s Republic of China (http://bzdt.ch.mnr.gov.cn/index.html, accessed on 8 December 2022).

The Belt and Road Initiative is an open and inclusive international regional economic cooperation network in which all willing interested countries can join, with no entirely closed spatial scope [1]. In this study, with reference to relevant studies and data availability [49], 74 countries including Russia, India and Myanmar are taken as the study area (Figure 1). The study area is divided into eight regions in Southeast Asia, South Asia, Central Asia, West Asia, China–Mongolia–Russia, Europe, nine African countries and Oceania. The political risk data were obtained from the World Bank database, the economic risk data were obtained from the World Bank database, the OECD database, the Knoema database and the Trend Economy database, and the energy risk data were obtained from the International Energy Agency and Eurostat. Ethnoreligious risk, military coup risk and external conflict data were obtained from the International Country Risk Guide, the violent armed threats data were obtained from the Fund for Peace website, and the terrorism threat data were obtained from the Institute for Economics and Peace’s Global Terrorism Index. According to the diplomatic relations level between countries and the United States and the signing of political, economic and military cooperation agreements, the relations between countries and the United States are divided into close, relatively close, general, relatively not close and hostile. At the same time, the Likert scale was used to assign values to the relations between countries and the United States, with a maximum score of 5 points and a minimum score of 1 point. The foreign debt burden data was obtained from the World Bank’s International Debt Statistics, the CEIC global database, the IMF database and the FRED database. The aid dependence data were obtained from the OECD database, and the Israel’s aid dependence data was obtained from the USAID Data Services. For the missing individual data in individual years, this study used multiple imputation method to fill in.

## 3. Evolution of Geopolitical Risk in Countries along the Belt and Road

### 3.1. Temporal Evolution of Geopolitical Risk in Countries along the Belt and Road

According to the entropy weight TOPSIS method for geopolitical risk measurement (Table 2), the polarization of geopolitical risk in countries along the Belt and Road was very significant, and the overall trend of geopolitical risk tended to deteriorate. From 2005 to 2020, the maximum geopolitical risk of countries along the Belt and Road reached 0.766, and the minimum geopolitical risk was 0.097, with the maximum risk being 7.9 times the minimum risk. High geopolitical risk countries were mainly located in the Middle East and South Asia, while low geopolitical risk countries were mainly located in the Europe and Oceania regions. Afghanistan, Iraq, Sudan, Syria, Pakistan, Ethiopia, Myanmar, Sri Lanka, Iran and Lebanon were the 10 countries with the highest average geopolitical risk along the Belt and Road, while New Zealand, Australia, Poland, Portugal, Czech Republic, Lithuania, Hungary, Slovenia, Italy and Croatia were the 10 countries with the lowest average geopolitical risk. Table 2 shows the chronological changes in geopolitical risk of countries along the Belt and Road. In terms of time, the average geopolitical risk level of countries along the Belt and Road from 2005 to 2020 has risen from 0.335 to 0.377, showing a slight upward trend in the overall geopolitical risk. In terms of countries, 59 countries along the Belt and Road saw their geopolitical risk level rise, with the number of countries with rising risk accounting for 79.73% of all countries, which indicates that the geopolitical risk situation of the countries along the Belt and Road tended to deteriorate.

It is worth noting that the Belt and Road Initiative is an important turning point in the evolution of geopolitical risk (Table 2). The average geopolitical risk of countries along the Belt and Road was 0.330 in 2005–2013, and the average geopolitical risk of countries along the Belt and Road was 0.356 in 2013–2020. After 2013, the geopolitical risk of 27 countries changed from negative growth to positive growth, which further indicates that the geopolitical risk situation of the countries along the Belt and Road tended to deteriorate. According to the geopolitical risk ranking in Table 2, the countries with geopolitical risk increases of more than 0.075 were Syria, Yemen, Libya, Thailand, Turkey, Egypt and Ireland in 2005–2013, and the countries with geopolitical risk increases of more than 0.08 were Myanmar, Yemen, Tunisia, Ukraine, Jordan, Germany and New Zealand in 2013–2020 (Table 2). The countries with the largest increases in geopolitical risk were Syria and Yemen, where geopolitical risk increased by a factor of 1.442 and 1.467, respectively. The multiple dilemmas of military conflict, ethnic strife, political unrest and economic stagnation brought about by the Syrian war and the Yemeni civil war spiked sovereign and military risk in both countries. Iraq was the country with the largest decline in geopolitical risk from 2005–2020, but Iraq’s geopolitical risk was always at a high risk level (Table 2). The geopolitical risk situation in Iraq has slowed down since the end of the Iraq War. However, the political turmoil caused by sectarian conflicts between Shiites and Sunnis, the military conflicts and armed threats brought about by the Kurdish independence movement and the frequent terrorist attacks by the “Islamic State” extremist organizations have all kept the geopolitical risk in Iraq high. It is worth noting that geopolitical risk in both Russia and Ukraine continued to rise after the 2013 Ukraine crisis, jumping from the medium risk level to the high risk level, which indicates that the political and economic security turmoil brought about by the Ukraine crisis has had a sustained impact on geopolitical risk in Russia and Ukraine.

**Figure 2:**
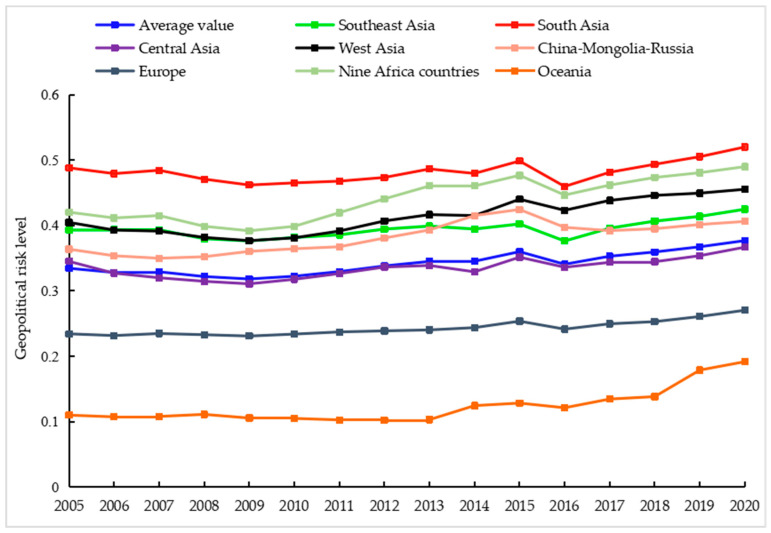
Changes in geopolitical risk in regions along the Belt and Road from 2005 to 2020.

From the regional perspective, the geopolitical risk situation along the Belt and Road was different. From 2005 to 2020, the average geopolitical risk in Oceania, Europe, Central Asia, China–Mongolia–Russia, Southeast Asia, West Asia, nine African countries and South Asia increased sequentially (Figure 2). The average geopolitical risk in Oceania hovered between 0.1 and 0.2, and Oceania was always at a very low risk level. Geopolitical risks in the European region were less volatile and showed a slight overall increase. Geopolitical risk in Europe remained stable from 2005 to 2013. From 2013 to 2020, Europe was affected by the refugee crisis, terrorist attacks, the Ukraine crisis and the COVID-19 epidemic, and geopolitical risk increased amid fluctuations. During this period, the Central and Eastern Europe region was deeply affected by the fierce geopolitical game of major powers, and the geopolitical risk increased significantly. In 2005 to 2020, the geopolitical risk level of Central Asia was in the medium risk level, with small fluctuations of geopolitical risk. Geopolitical risk in China, Mongolia and Russia rose and then fell, and 2015 was a turning point in the evolution of regional geopolitical risk. China’s rapid economic growth from 2005–2015 and international energy price volatility increased economic risk in Russia and Mongolia, while geopolitical games between Russia, the United States and Europe and external intervention in Mongolia by the United States, Europe and Japan tended to increase geopolitical risk in the China–Mongolia–Russia region during this period. In 2015–2018, the recovery of economic growth in Russia and Mongolia and the strengthening of regional cooperation between China, Mongolia and Russia injected “living water” into regional security and stability, contributing to a decline in overall geopolitical risk in the China–Mongolia–Russia region. In 2018–2020, the trade friction between China and the United States, the escalation of the confrontation between the United States and Russia and the impact of the COVID-19 epidemic make the geopolitical risk in the China–Mongolia–Russia region rise again. Southeast Asia’s geopolitical risks were less variable from 2005 to 2015. However, due to frequent terrorist attacks and violent threats from antigovernment forces, the geopolitical risks of Southeast Asia were rising rapidly from 2016–2020. Influenced by the Arab Spring, the Syrian War, the rise of the “Islamic State”, the Yemeni civil war and the COVID-19 epidemic, the geopolitical risk in West Asia rose rapidly in the two periods of 2009–2013 and 2016–2020. Geopolitical risk fluctuated widely in nine African countries. Geopolitical risk declined in nine African countries during 2005–2009. From 2009 to 2015, under the impact of the international financial crisis and the rapid spread of the Arab Spring, the geopolitical risk of nine African countries increased significantly. From 2015 to 2016, with the recovery of economic growth, the reduction in the foreign debt burden and the relative reduction in external intervention, the geopolitical risk of nine African countries decreased significantly. In 2016–2020, influenced by the intervention of major powers, the recovery of terrorism and the spillover effect of the Syrian war, Tunisia and Egypt were in a state of turmoil, and Libya’s turmoil continued to deteriorate, leading to the continued volatility and rise in geopolitical risk in nine African countries. Due to complex sectarian conflicts, the resistance of the northeastern states against the central government’s Hindu nationalism, and the expansion of separatist terrorism in Kashmir, the geopolitical risk in South Asia has always been at a high level. It is worth noting that due to the impact of the downturn in world economic development and the outbreak of the COVID-19 epidemic, all regions are experiencing a significant rise in geopolitical risk evident in 2018–2020. Since 2018, the rise in nationalism and populism, intensified military competition and intense political struggle in West Asia, North Africa, Europe and South Asia have significantly worsened the geopolitical security situation along the Belt and Road, and the geopolitical risk has increased significantly.

### 3.2. Spatial Evolution of Geopolitical Risk in Countries along the Belt and Road

**Figure 3:**
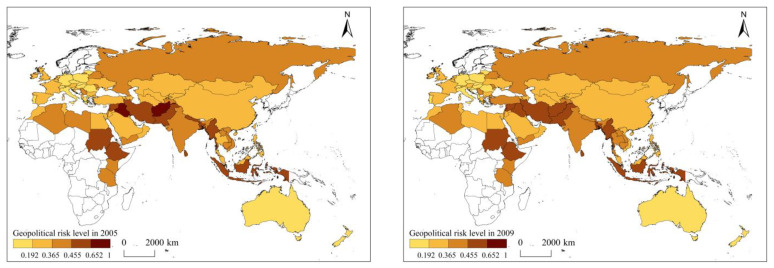

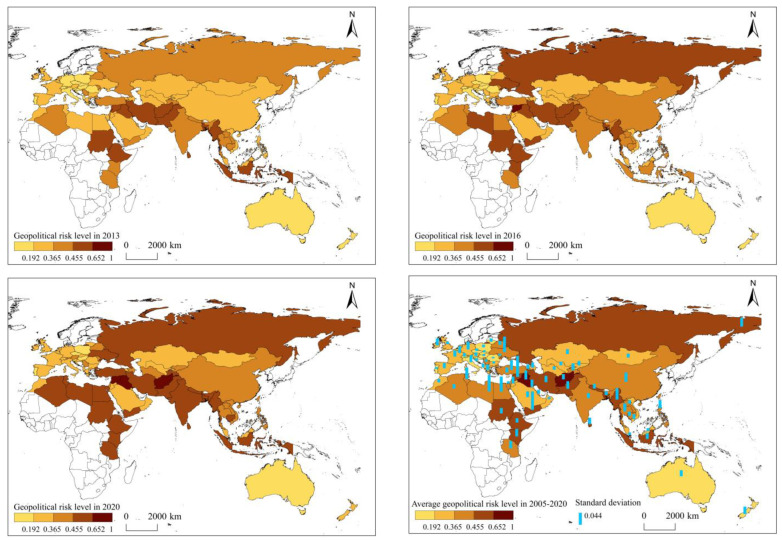
Spatial pattern of geopolitical risk in countries along the Belt and Road. Note: The map is drawn to the standard map of GS (2016) 1667 that comes from the standard map service system of the ministry of natural resources of the people’s Republic of China (http://bzdt.ch.mnr.gov.cn/index.html, accessed on 8 December 2022).

Geopolitical risk along the Belt and Road has significant agglomeration characteristics in spatial distribution (Figure 3). The Middle East and Eastern Europe are high geopolitical risk agglomeration regions, while Oceania and Central and Western Europe are low-risk agglomeration areas. High geopolitical risk areas are concentrated in the Middle East and Eastern Europe, with Afghanistan, Iraq, Ukraine and Russia being the main high-risk concentration centers. According to the gradient of risk concentration, the geopolitical risk belt in the Middle East has significant gradient characteristics, showing that Afghanistan and Iraq constitute two high-risk peaks, Syria, Iran and Pakistan form secondary high-risk areas, and Yemen, Israel, Turkey and Tajikistan form third level high-risk areas in the periphery. The threat of armed violence brought about by the wars in Iraq and Afghanistan, combined with political turmoil, ethnic and religious conflicts and a high incidence of terrorist attacks, has made Iraq and Afghanistan the high-risk centers of the high-risk belt in the Middle East. The geopolitical risk belt in Eastern Europe also has gradient characteristics, showing that Russia and Ukraine are high-risk centers, and Belarus and Moldova form secondary high-risk areas. The United States’ containment of Russia, the energy exports dependence and the Russia-Ukraine conflict made Russia’s geopolitical risk always high. Stimulated by ethnic conflicts, political turbulence and threats of violent armed forces brought about by the intervention of major powers and the Ukraine crisis, Ukraine’s geopolitical risk rose rapidly from 0.320 to 0.456, becoming another high-risk center in Eastern Europe. The geopolitical risk of the nine African countries is centered on Sudan and Ethiopia as high-risk center, and Algeria, Libya and Kenya constitute secondary high-risk areas. The division and ongoing conflicts between North and South Sudan have kept Sudan’s geopolitical risk at a very high risk level, while the Tigrayan civil war, Western economic sanctions, refugee crisis and food crisis have made Ethiopia a high geopolitical risk center in East Africa. It is worth noting that military conflicts, terrorist attacks, ethnic conflicts and economic fluctuations in high geopolitical risk areas have the characteristic of “one hair moves the whole body”, and such regional high-risk centers as Afghanistan, Iraq, Sudan, Ethiopia, Ukraine and Russia have obvious risk spillover effects. In 2005–2013, the geopolitical risk spillover brought by the Afghanistan war and the Iraq war significantly increased the geopolitical risk of neighboring countries such as Iran, Lebanon, Turkey and Algeria. In 2005–2020, the outbreak of the Ukraine crisis made the average geopolitical risk level of Eastern European countries rise from 0.330 to 0.392. This indicates that there are several high geopolitical risk centers along the Belt and Road, and there is a certain degree of geopolitical risk spillover. In addition, the standard deviation of geopolitical risk in Iraq, Syria, Yemen and other countries exceeded 0.038 (Figure 3), which indicated that the geopolitical risk in some countries with high geopolitical risk fluctuated greatly.

Low risk areas also have certain agglomeration characteristics. Oceania and central and western Europe are two major extremely low geopolitical risk agglomeration areas. In Central and Western Europe, the geopolitical risk of Poland, Portugal, the Czech Republic, Hungary and Slovenia have always been at a very low risk level from 2005 to 2020. The geopolitical risk of Lithuania, Italy, Croatia, Switzerland, Romania, Slovakia, Germany and Albania, which are peripheral to the very low risk countries, have always hovered between very low risk and low risk. The geopolitical risk of Bulgaria, Belgium, Greece, Spain, the Netherlands, the United Kingdom, France, Austria, Ireland, which are marginal to very low risk countries, have been low in most years. These make the gradient agglomeration of very low geopolitical risk and low geopolitical risk extremely significant in Central and Western Europe. The geopolitical risk fluctuations of low geopolitical risk countries are polarized. The standard deviation of geopolitical risk in Romania, the Netherlands, Hungary, Slovenia, Portugal, Slovakia, Moldova, Bulgaria and Oman are all below 0.010, while the standard deviation of geopolitical risk in New Zealand, Germany, Ireland and France all exceeds 0.026. Most of the countries in Central and Western Europe are NATO allies of the United States. They are politically stable and economically developed, with low foreign trade dependence degree and foreign investment degree. Their military risks and economic risks are lower than those of other regions along the Belt and Road, making them a cluster of low geopolitical risks. In 2005–2020, the geopolitical risk of Australia and New Zealand in Oceania was mostly in the very low risk level, and only New Zealand rose to the low risk level in 2020 due to the rising economic risk caused by the COVID-19 epidemic. Australia and New Zealand are far away from global geopolitical conflict areas, politically stable, economically developed and have almost no external military threats, and thus constitute the most prominent low-risk geopolitical areas along the Belt and Road.

## 4. Analysis of Geopolitical Risk Obstacle Factors in Countries along the Belt and Road

In order to comparatively analyze the evolution of geopolitical risk obstacle factors in countries along the Belt and Road, the three indicators with the most significant obstacle factors were analyzed in 2005, 2009, 2013, 2016 and 2020 (Table 3). As shown in Table 3, geopolitical risk factors along the Belt and Road varied greatly in different years, and geopolitical risk incentives tended to be decentralized and complex as a whole. In 2005, the global terrorism index had the most significant role in the close relations with the United States, and its contribution to the geopolitical risk of countries along the Belt and Road was about 36.48%, which was the top obstacle factor for 27 countries along the Belt and Road. In 2009, the role of the global terrorism index in the geopolitical risk of the Belt and Road declined, while the number of countries as the top obstacle factor and the proportion of the top three obstacle factors, such as close relations with the United States, foreign trade dependence degree, military intervention in politics intensity and foreign debt burden increased, and the role of the global terrorism index in the geopolitical risk of the Belt and Road increased. In 2013, both foreign debt burden and aid dependence were the top obstacle factors for 14 countries, and both became the most important obstacle factors for geopolitical risk in countries along the Belt and Road. In 2016, the number of countries relying on aid dependence as the top obstacle factor decreased by nine, while close relations with the United States and the foreign debt burden became the most prominent top obstacle factors, respectively, in 17 and 15 countries. In 2020, the top geopolitical risk factors of countries along the Belt and Road changed significantly. The number of countries with political stability, energy dependence degree, ethnic conflict intensity, religious conflict intensity, military intervention in politics intensity, global terrorism index and extern debt burden as the top three obstacle factors increased significantly, and close relations with the United States became the top obstacle factor with the most occurrence. Overall, the contribution of the global terrorism index and close relations with the United States decreased, while the contribution of the military intervention in politics intensity, foreign trade dependence degree, energy dependence degree and foreign debt burden increased, and the geopolitical risk triggers of countries along the Belt and Road tended to be diversified and complicated.

By analyzing the changes in geopolitical risk obstacle factors in various regions in Table 3, it was found that the regional characteristics of geopolitical risk obstacle factors for countries along the Belt and Road were significant. The high frequency of two indicators of the military intervention in politics intensity (A_7_) and close relations with the United States (A_11_) were the most important obstacle factors in Southeast Asia (Table 3). The military was the most powerful force in the political lives of Myanmar, Thailand, Cambodia and other Southeast Asian countries, and the military controls the power in some countries. The “Indo-Pacific strategy” of the United States takes Southeast Asia as an important strategic pivot point and intervenes in Southeast Asia’s regional affairs, making the geopolitical security situation in Southeast Asia deteriorate. The global terrorism index (A_9_) and military intervention in politics intensity (A_7_) had an impact on all countries in the South Asia region, and the frequency of the global terrorism index as the top obstacle factor was much higher than other indicators, constituting a key risk predisposing factor in South Asia (Table 3). The combination of limited government capacity and widespread dissatisfaction with corruption, ethnoreligious policies and socioeconomic marginalization has made South Asia an attractive base of operations for terrorist groups, and the spread of religious extremism and ethnic radicalism has made South Asia one of the most serious areas of terrorism along the Belt and Road. The political, economic and social development of Pakistan, Afghanistan and Sri Lanka are deeply influenced by the military, and military intervention in politics seriously affects the political stability of the countries and becomes one of the important factors inducing geopolitical risk. The main obstacle factors for geopolitical risk in Central Asian countries are the intensity of religious conflicts (A_6_), the intensity of violent armed threats (A_8_) and close relations with the United States (A_11_) (Table 3). Complex ethnic relations and rapid development of religious extremism have led to frequent large-scale religious conflicts and armed conflicts in Central Asia. As the center of the heartland, Central Asia is the key area for the competition between the United States and Russia, resulting in the geopolitical security of Central Asia is deeply affected by the great power game. The main obstacle factors of geopolitical risk in West Asia are the external conflict intensity (A_10_), military intervention in politics intensity (A_7_) and global terrorism index (A_9_) (Table 3). On the one hand, disputes over hydrocarbon and water resources in West Asia have led to conflicts and frictions over resources in the region, while weak state governance and the prevalence of extremist religious ideology have led to frequent terrorist attacks. On the other hand, the rich resources and special geopolitical location also make West Asia an important place for great power games. The superposition of intraregional conflicts and extraterritorial major power intervention make the geopolitical security of West Asia very fragile. The main obstacle factor of geopolitical risk for China and Russia is the global terrorism index (A_9_), and the foreign debt burden (A_12_) and aid dependence (A_13_) are the main obstacle factors of geopolitical risk for Mongolia (Table 3). The threat of jihadists in the Caucasus, Chechen terrorism and “East Turkistan” terrorists are the most destabilizing factors for the geopolitical security of Russia and China. Mongolia, whose economic development is relatively dependent on foreign debt and foreign aid, is vulnerable to intervention by major powers. The main obstacle factors of geopolitical risk in European countries vary greatly. The main obstacle factors of the geopolitical risk in Eastern European countries are close relations with the United States (A_11_) and the intensity of external conflicts (A_10_), the main obstacle factor of the geopolitical risk in Western European countries are foreign debt burden (A_12_), and the main obstacle factors of the geopolitical risk in Central and Southern European countries are energy dependence degree (A_4_) and foreign debt burden (A_12_) (Table 3). Eastern European countries are at the forefront of the NATO camp to encircle Russia. The geopolitical risk of Eastern European countries continues to rise under the influence of the intense America–Russia game, and the Ukraine crisis has intensified external conflicts in Eastern Europe, pushing the geopolitical risk in the region to rise further. Economic imbalances and worsening fiscal deficits in Western, Central and Southern European countries have led to the outbreak of the European debt crisis and a sharp expansion of foreign debt, leading to increased economic and political risk, while the dependence of Western, Central and Southern European countries on energy imports, especially from Russia, is exacerbating the energy risk in these regions. The main obstacle factors of nine African countries are close relations with the United States (A_11_) and the military intervention in politics intensity (A_7_) (Table 3). The frequent American involvement in the affairs of African countries and military operations involving Africa have seriously threatened Africa’s geopolitical security, while frequent military coups and a high incidence of military conflicts have kept the geopolitical risk in relatively backward Africa at a high level for a long time. Although the geopolitical risk in Australia and New Zealand is low, in the two countries, as typical postcolonial immigrant countries with prevalent multiculturalism, ethnic conflicts brought about by the lack of national identity (A_5_) are becoming an important factor triggering the geopolitical risk of both countries (Table 3).

**Figure 4:**
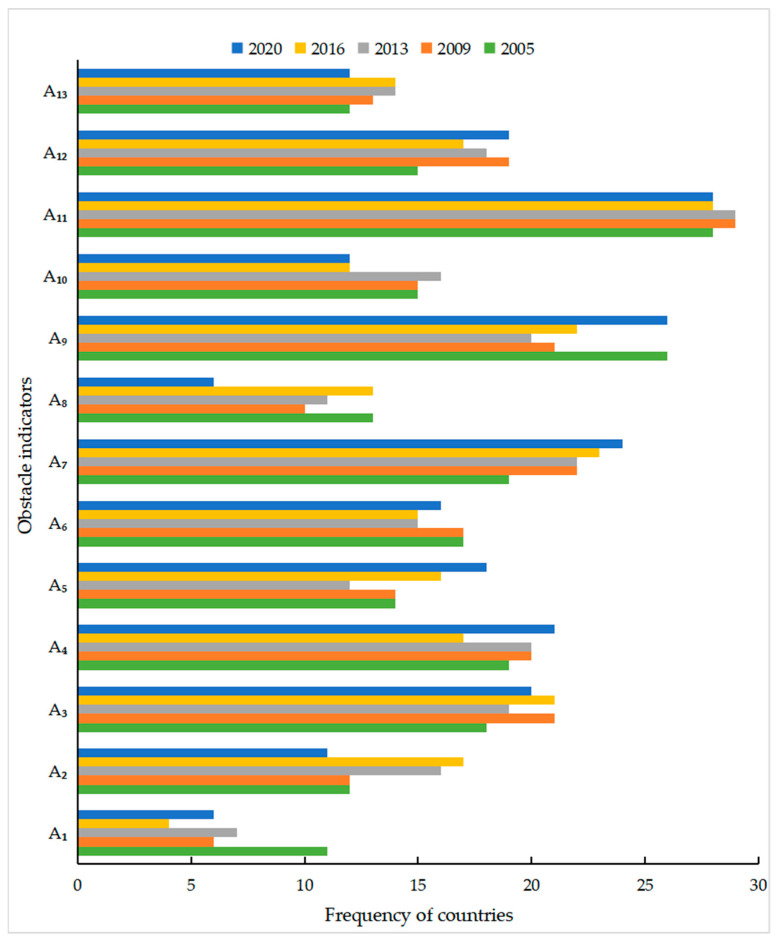
Frequency distribution of geopolitical risk obstacle indicators in countries along the Belt and Road.

According to the frequency distribution of indicators of geopolitical risk obstacle factors (Figure 4), terrorism and close relations with the United States are the most important obstacle factors of geopolitical risk of countries along the Belt and Road, while military intervention politics, foreign trade dependence and foreign debt burden are important obstacle factors of the geopolitical risk of countries along the Belt and Road. In order to further explore the main obstacle factors of geopolitical risk in the countries along the Belt and Road, this study used 13 geopolitical risk indicators to create a frequency distribution chart (Figure 4). The total frequencies of close relations with the United States (A_11_), the global terrorism index (A_9_), the military intervention in politics intensity (A7), foreign trade dependence degree (A_3_) and energy dependence degree (A_4_) were 146, 118, 112, 99 and 97, respectively, which were the four obstacle factors with the highest frequency of occurrence. The frequency of the close relations with the United States and the global terrorism index exceeded 20 in 2005, 2009, 2013, 2016 and 2020, and they were the top obstacle factor for 40 countries, including Thailand, Myanmar, Philippines, Vietnam and Laos. These indicated that the close relations with the United States and the global terrorism index were the primary causes of the outbreak of geopolitical risk in countries along the Belt and Road. The frequency of military intervention in politics increased from 20 in 2005 to 24 in 2020, which became a secondary obstacle factor for the outbreak of geopolitical risks in countries along the Belt and Road. Military intervention in politics has made some countries along the Belt and Road backward democratization, corruption, political instability and ethnic and ethnoreligious issues unable to be effectively solved, thus increasing geopolitical risk,. The frequency of foreign trade dependence degree and energy dependence degree fluctuated around 20 in 2005, 2009, 2013, 2016 and 2020. The excessive dependence on foreign trade and energy makes the economic sovereignty and energy security of some developing countries along the Belt and Road unable to be effectively guaranteed. Therefore, to prevent and control geopolitical risk along the Belt and Road, terrorism and the close relations with the United States should be addressed as the most critical geopolitical risk factors, while paying attention to important geopolitical risk obstacle factors such as the military intervention in politics intensity, trade dependence degree and foreign debt burden.

## 5. Classification of Geopolitical Risk Types in Countries along the Belt and Road

**Figure 5:**
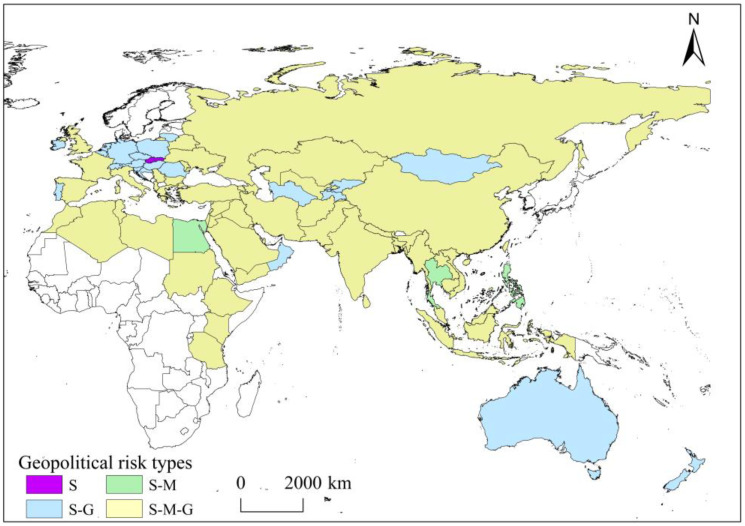
Geopolitical risk types of countries along the Belt and Road. Note: The map is drawn to the standard map of GS (2016) 1667 that come from the standard map service system of the ministry of natural resources of the people’s Republic of China (http://bzdt.ch.mnr.gov.cn/index.html, accessed on 8 December 2022).

The identification of risk obstacle factors reveals the main factors of geopolitical risk in each country, but does not identify geopolitical risk types along the Belt and Road. In combination with the impact of the three subsystems of sovereign risk, military risk and major power intervention on geopolitical risk, this study uses the minimum variance method to divide the geopolitical risk of countries along the Belt and Road into single system risks, double system risks and three system risks. Single system risk includes sovereign risk dominant type (S), and double system risk includes sovereign and military risk dominant type (S-M) and sovereign and major power intervention risk dominant type (S-G). Three system risks are sovereign and military and major power intervention risk jointly dominated type (S-M-G) (Figure 5).

The single system risks include only the S type, with a very small distribution of only one country, Slovakia (Figure 5). The risk of military and major power intervention is low in Slovakia, but as a multiethnic, multiparty country, ethnic conflicts between ethnic Slovaks and Hungarians and the ethnic animosity they bring are serious threats to Slovak social stability, and the intertwining of complex party relations with nationalist issues further aggravates the political situation and social turbulence of Slovakia. In addition, Slovakia’s economic development is very dependent on EU trade and capital, with EU member states accounting for about 80% of its import and export trade in goods, and this excessive dependence on the single market prevents Slovakia’s economic sovereignty from being fully guaranteed. The combination of political, economic and ethnic risk causes Slovakia’s sovereign risk to dominate the geopolitical risk.

The double system risks include the S-M type and S-G type, which cover 25 countries, with Central and Eastern Europe having the highest concentration of them (Figure 5). The S-M type countries are Thailand, the Philippines and Egypt. On the one hand, Thailand, the Philippines and Egypt are characterized by intense partisan political struggles and corrupt officials in politics, high foreign trade dependence degree and investment dependence degree in the economy and persistent ethnic conflicts in the three southern provinces of Thailand and Christian and Muslim conflicts in Egypt, making sovereignty risk one of the main aspects of geopolitical risk in these three countries. On the other hand, the military in Thailand, the Philippines and Egypt are extremely influential in the political life of the country, and the intensity of military intervention in politics is greater, while the radical Islamists in Egypt, antigovernment forces in the Philippines and ultranationalist forces in Thailand make the three countries face greater pressure of violent armed threats and terrorist threats, and also causing military risk to become another major aspect of geopolitical risk in the three countries. The S-G type is mainly located in Ukraine, Poland, Singapore, Kyrgyzstan, Mongolia and the other 22 countries. In terms of sovereignty risk, these countries are economically more dependent on a single market or investment from a few allies, and most of them are more dependent on neighboring energy powers in energy. In terms of politics and military affairs, they are deeply influenced by the United States and Russia, and it is difficult for them to maintain complete neutrality in the major power intervention or the great power game. 

The S-M-G type is distributed among 42 developing countries in Asia and Africa, including Indonesia, Myanmar, Vietnam, Malaysia, Laos, Cambodia and Nepal, and six developed countries, including Israel, Italy, France, Spain, the United Kingdom and Greece. It accounts for 64.86% of the countries in the study area and is the most important geopolitical risk type for countries along the Belt and Road (Figure 5). Although the geopolitical risk dynamics of the countries of the three systemic risks are not the same, the degree of the contribution of sovereign risk, military risk and major power intervention risk to the changes in geopolitical risk in these countries is more balanced, and a single subsystem cannot dominate, causing the geopolitical risk of these countries to be dominated by a combination of sovereign risk, military risk and major power intervention. In terms of sovereignty risk, the fierce party struggle of Bangladesh, Libya, Sri Lanka and other countries brings political instability. Vietnam, Malaysia, Kazakhstan and other countries’ excessive foreign trade dependence and investment dependence leads to a high economic risk. In China, Jordan, Albania, Bulgaria, Ukraine and Morocco and other countries with a stronger energy dependence degree, the energy risk cannot be ignored. In Myanmar, Laos, Pakistan, India and other countries with fierce ethnic and religious conflicts, there is a high ethnic and religious risk. In terms of military risk, Myanmar, Pakistan, Indonesia and Sri Lanka have a high intensity of military intervention in politics. Libya, Kazakhstan, Uzbekistan and Yemen are troubled by the violent armed threat of antigovernment forces. Iraq, Afghanistan, Pakistan, India and Indonesia are deeply threatened by terrorism. In terms of the risk of major power intervention, Iran, Saudi Arabia, Lebanon and Turkey are deeply affected by external conflicts such as wars, ethnic conflicts and religious issues in neighboring countries. The economic and political and military development of Vietnam, Myanmar, Iraq, Syria and Saudi Arabia are deeply affected by the interference of the United States. Kazakhstan, Sri Lanka and Bangladesh are under the heavy pressure of foreign debts. Egypt, Laos, Cambodia and Nepal are highly dependent on aid dependence, which impacts their internal and foreign affairs. It is worth noting that some S-M-G type geopolitical risk countries such as Myanmar, Iran and Yemen have formed a linkage response, with sovereign risk, military risk and major power interventions interacting and jointly driving national geopolitical risk to move higher. Britain, Israel, Italy, France, Spain and Greece have a certain degree of trade dependence and energy dependence in terms of sovereignty risk. At the same time, the transition of democracy and party struggle are impacting political stability, and the terrorist attacks caused by the resurgence of extremism are seriously threatening their national security. In addition, as allies of the United States, their geopolitical practices are to a large extent dependent on the global hegemony of the United States, and their geopolitical risk are jointly dominated by sovereign risk, military risk and major power intervention.

## 6. Conclusions

This study constructs a multidimensional geopolitical risk assessment index system, analyzes the spatial and temporal evolution of geopolitical risk in countries along the Belt and Road, analyzes the geopolitical risk obstacle factors in countries along the Belt and Road, and classifies geopolitical risk types in countries along the Belt and Road. The results of this study showed that the polarization of geopolitical risk in countries along the Belt and Road was very significant, and the overall trend of geopolitical risk tended to deteriorate. The Middle East and Eastern Europe were the most important geopolitical risk zones along the Belt and Road, and Afghanistan, Iraq, Russia and Ukraine were the main high-risk centers, with significant risk spillover effects from high-risk geopolitical centers. The contribution of the geopolitical risk factors of countries along the Belt and Road tended to be decentralized and complicated. Terrorism and close relations with the United States were the most important obstacle factors of the geopolitical risk of countries along the Belt and Road, while military intervention politics, trade dependence and foreign debt burden were important obstacle factors of the geopolitical risk of countries along the Belt and Road. Geopolitical risk along the Belt and Road can be divided into sovereign risk dominant type, sovereign and military risk dominant type, sovereign and major power intervention risk dominant type, and sovereign and military and major power intervention risk jointly dominated type, among which sovereign and military and major power intervention risk jointly dominated type was the most important geopolitical risk type.

The empirical assessment of geopolitical risk along the Belt and Road showed that the geopolitical risk along the Belt and Road were not optimistic, and the scientific and reasonable response, prevention and control of geopolitical risk were extremely critical to promoting the Belt and Road. Based on the analysis of geopolitical risk evolution, obstacle factors and risk types along the Belt and Road, we believe that the following four aspects are especially essential. First, relevant departments should enhance geopolitical risk awareness and strengthen the monitoring and prediction of geopolitical risk along the Belt and Road. While strengthening geopolitical risk awareness, relevant functional departments should strengthen geopolitical risk assessment and risk factor monitoring along the Belt and Road, establish a geopolitical risk prediction and prevention mechanism, construct an effective multilateral crisis and emergency management system, strengthen post-event response and disposal capabilities and reduce risk losses. Second, it is important to pay attention to the most critical factors of terrorism and close relations with the United States, as well as important geopolitical risk factors such as the military intervention in politics intensity, trade dependence degree and foreign debt burden. As the most critical geopolitical risk triggers for countries along the Belt and Road, terrorism and close relations with the United States should be the focus of geopolitical risk prevention and control, while the military intervention in politics intensity, trade dependence degree and foreign debt burden will play a more important role in the geopolitical game in the post-COVID-19 era. Third, the control of regional geopolitical risk spillover should be strengthened. The spillover effect of high geopolitical risk areas along the Belt and Road is obvious, and the risk spillover of individual high geopolitical risk countries may trigger the “domino effect” of regional geopolitical risk. It is necessary to make comprehensive use of international political consultation, geo-economic cooperation and the building of a community of human destiny to reduce the spillover of geopolitical risk. Fourth, we should develop reasonable risk prevention and control programs according to different types of geopolitical risk. There are various types of geopolitical risk along the Belt and Road, and different types of geopolitical risk do not have the same focus of prevention and control and key elements of monitoring. Formulating corresponding response scheme based on geopolitical risk types can not only improve the applicability of the scheme to deal with the geopolitical risk, but also improve the effectiveness of the scheme.

As a complex evolutionary system, the quantitative assessment of geopolitical risk is the key support for the scientific judgment of geo-setting situations. Unlike geopolitical risk assessment from a single political, economic or social perspective [50], this study emphasizes that the core of geopolitical risk is sovereign and military security, and its risk assessment should integrate multiple sovereign risk elements, military risk elements and major power intervention elements for dynamic analysis. Just as Liu’s study believed that the political risk level in Central and West Asia was high [51], this paper also found that the geopolitical risk in Central and West Asia was high. However, political risk cannot be completely equated with geopolitical risk. The changing trend of geopolitical risk in countries along the Belt and Road was opposite to that of political risk. Global terrorism, close relations with the United States and military intervention in politics play a more significant role in geopolitical risks. This study did not discuss the prediction of geopolitical risk, the impact effects of geopolitical risk and the evolution mechanism of geopolitical risk. In the future, we should strengthen the research on the prediction geopolitical risk, the impact effects of geopolitical risk and the evolution mechanism of geopolitical risk.

## Figures and Tables

**Table 1:** Geopolitical risk indicator system of countries along the Belt and Road.

Target Layer	Element Layer	Indicator Layer	Indicator Name	Indicator Direction
Geopolitical risk in countries along the Belt and Road	Sovereign risk (S)	Political Risk	Political stability(A_1_)	−
		Economic Risk	Investment dependence degree (A_2_)	+
			Foreign trade dependence degree(A_3_)	+
		Energy Risk	Energy dependence degree (A_4_)	+
		Ethnoreligious risk	Ethnic conflict intensity (A_5_)	+
			Religious conflict intensity (A_6_)	+
	Military risk (M)	Military coup risk	Military intervention in politics intensity (A_7_)	+
		Violent armed threats	Violent armed threat intensity (A_8_)	+
		Terrorism Threat	Global terrorism index (A_9_)	+
	Major power intervention (G)	External Conflict	External conflict intensity (A_10_)	+
		Alliances	Close relations with the United States (A_11_)	+
		Foreign debt burden	Foreign debt stock as a percentage of GNI(A_12_)	+
		Aid dependence	Net official development assistance received as a percentage of GNI (A_13_)	+

Note: + indicates that the indicator is positively correlated with geopolitical risk, and − indicates that the indicator is negatively correlated with geopolitical risk.

**Table 2:** Evolution of geopolitical risk level of countries along the Belt and Road.

Country	2005	Rank	2009	Rank	2013	Rank	2016	Rank	2020	Rank
Afghanistan	0.690	2	0.631	1	0.642	2	0.640	2	0.666	2
Iraq	0.765	1	0.602	2	0.645	1	0.620	3	0.653	3
Sudan	0.607	3	0.573	3	0.627	3	0.600	4	0.551	8
Syria	0.468	12	0.457	11	0.610	4	0.669	1	0.675	1
Pakistan	0.524	6	0.509	4	0.545	5	0.545	5	0.603	5
Ethiopia	0.523	7	0.486	6	0.528	6	0.527	6	0.544	9
Myanmar	0.528	5	0.462	9	0.498	10	0.488	8	0.614	4
Sri Lanka	0.533	4	0.505	5	0.511	9	0.437	18	0.537	10
Iran	0.475	11	0.473	7	0.516	7	0.462	12	0.568	6
Lebanon	0.460	13	0.461	10	0.512	8	0.480	9	0.522	12
Bangladesh	0.501	9	0.457	12	0.473	12	0.458	13	0.487	17
Nepal	0.484	10	0.439	13	0.469	15	0.446	17	0.506	14
Yemen	0.382	25	0.382	23	0.461	16	0.519	7	0.560	7
Indonesia	0.520	8	0.468	8	0.473	13	0.417	22	0.473	21
Kenya	0.432	17	0.421	14	0.478	11	0.456	14	0.524	11
Russia	0.435	16	0.411	17	0.453	17	0.473	10	0.513	13
Algeria	0.437	15	0.414	15	0.446	18	0.420	21	0.487	18
Libya	0.367	27	0.335	34	0.471	14	0.471	11	0.501	16
Israel	0.438	14	0.413	16	0.419	26	0.410	25	0.442	28
Thailand	0.338	33	0.393	21	0.440	19	0.449	16	0.444	27
India	0.398	21	0.399	19	0.433	21	0.413	24	0.466	22
Cambodia	0.402	20	0.395	20	0.427	22	0.406	26	0.461	24
Laos	0.416	18	0.401	18	0.424	24	0.371	32	0.411	32
Tanzania	0.408	19	0.367	28	0.422	25	0.402	29	0.484	19
Turkey	0.320	40	0.376	24	0.426	23	0.435	19	0.458	25
Tajikistan	0.396	22	0.370	27	0.409	28	0.405	27	0.475	20
Tunisia	0.351	31	0.339	33	0.409	29	0.416	23	0.504	15
Egypt	0.334	35	0.298	43	0.434	20	0.432	20	0.466	23
Belarus	0.366	28	0.371	26	0.412	27	0.363	34	0.411	31
Moldova	0.381	26	0.391	22	0.389	32	0.372	31	0.410	33
Philippines	0.345	32	0.345	32	0.393	31	0.392	30	0.433	29
China	0.331	37	0.363	29	0.403	30	0.404	28	0.415	30
Serbia	0.385	24	0.372	25	0.385	33	0.359	35	0.402	34
Ukraine	0.320	41	0.315	39	0.352	36	0.455	15	0.456	26
Kyrgyzstan	0.361	30	0.333	35	0.362	34	0.369	33	0.398	35
Vietnam	0.390	23	0.362	30	0.332	40	0.321	40	0.374	37
Azerbaijan	0.335	34	0.327	36	0.361	35	0.358	36	0.388	36
Turkmenistan	0.333	36	0.316	38	0.338	38	0.332	38	0.368	39
Armenia	0.319	42	0.311	40	0.332	39	0.344	37	0.363	40
Uzbekistan	0.363	29	0.324	37	0.328	42	0.318	41	0.318	47
Ireland	0.263	54	0.349	31	0.345	37	0.313	43	0.304	48
Morocco	0.324	39	0.294	44	0.330	41	0.294	46	0.349	41
Austria	0.306	44	0.309	41	0.315	45	0.306	44	0.369	38
Mongolia	0.325	38	0.306	42	0.324	44	0.314	42	0.290	51
Saudi Arabia	0.316	43	0.275	46	0.285	46	0.326	39	0.335	42
Malaysia	0.303	45	0.289	45	0.327	43	0.284	48	0.320	46
Qatar	0.294	47	0.267	49	0.283	47	0.272	49	0.329	44
France	0.272	52	0.241	53	0.268	50	0.304	45	0.328	45
Singapore	0.295	46	0.270	48	0.279	48	0.259	53	0.293	49
Oman	0.268	53	0.254	51	0.277	49	0.258	54	0.291	50
Jordan	0.280	49	0.223	55	0.258	52	0.286	47	0.334	43
United Kingdom	0.286	48	0.271	47	0.258	51	0.249	56	0.288	52
Netherlands	0.250	56	0.252	52	0.243	55	0.260	51	0.261	56
Spain	0.277	50	0.256	50	0.220	58	0.201	59	0.258	58
Kazakhstan	0.273	51	0.211	56	0.257	53	0.257	55	0.275	54
Greece	0.192	61	0.239	54	0.247	54	0.235	57	0.284	53
Kuwait	0.260	55	0.197	58	0.221	57	0.268	50	0.245	59
Belgium	0.216	58	0.207	57	0.208	59	0.259	52	0.271	55
Bulgaria	0.207	59	0.195	59	0.224	56	0.195	60	0.200	65
Albania	0.224	57	0.181	60	0.187	62	0.175	66	0.206	64
Germany	0.174	65	0.166	64	0.165	67	0.225	58	0.260	57
Slovakia	0.185	62	0.178	61	0.189	60	0.189	61	0.209	63
Romania	0.181	63	0.178	62	0.188	61	0.175	65	0.199	66
Switzerland	0.164	66	0.169	63	0.187	63	0.181	62	0.230	61
Croatia	0.201	60	0.165	65	0.173	65	0.176	64	0.193	68
Italy	0.175	64	0.154	68	0.185	64	0.177	63	0.238	60
Slovenia	0.160	67	0.162	67	0.166	66	0.160	68	0.185	69
Hungary	0.153	68	0.163	66	0.165	68	0.167	67	0.184	70
Lithuania	0.147	70	0.147	69	0.151	69	0.152	70	0.194	67
Czech Republic	0.150	69	0.143	71	0.144	71	0.158	69	0.166	72
Portugal	0.130	72	0.143	70	0.151	70	0.142	71	0.154	73
Poland	0.130	71	0.109	72	0.118	72	0.125	73	0.151	74
Australia	0.115	73	0.106	73	0.099	74	0.134	72	0.174	71
New Zealand	0.105	74	0.105	74	0.107	73	0.108	74	0.210	62

**Table 3:** Major geopolitical risk obstacle factors of countries along the Belt and Road.

Country	2005			2009			2013			2016			2020		
	1	2	3	1	2	3	1	2	3	1	2	3	1	2	3
Thailand	A_9_	A_3_	A_5_	A_9_	A_6_	A_5_	A_9_	A_7_	A_6_	A_7_	A_9_	A_6_	A_7_	A_9_	A_6_
Indonesia	A_6_	A_9_	A_7_	A_6_	A_7_	A_11_	A_6_	A_5_	A_7_	A_6_	A_7_	A_5_	A_6_	A_7_	A_5_
Myanmar	A_7_	A_11_	A_9_	A_7_	A_11_	A_10_	A_7_	A_13_	A_11_	A_7_	A_11_	A_8_	A_11_	A_7_	A_9_
Philippines	A_9_	A_6_	A_1_	A_9_	A_1_	A_6_	A_9_	A_8_	A_7_	A_9_	A_8_	A_7_	A_9_	A_8_	A_7_
Singapore	A_12_	A_3_	A_4_	A_12_	A_3_	A_4_	A_12_	A_3_	A_2_	A_12_	A_3_	A_2_	A_12_	A_3_	A_2_
Vietnam	A_11_	A_7_	A_8_	A_11_	A_7_	A_3_	A_3_	A_7_	A_11_	A_3_	A_7_	A_11_	A_3_	A_7_	A_11_
Malaysia	A_3_	A_11_	A_8_	A_3_	A_11_	A_8_	A_3_	A_11_	A_8_	A_3_	A_8_	A_6_	A_3_	A_6_	A_5_
Laos	A_13_	A_11_	A_8_	A_13_	A_11_	A_6_	A_11_	A_7_	A_13_	A_7_	A_6_	A_11_	A_7_	A_13_	A_6_
Cambodia	A_11_	A_13_	A_3_	A_11_	A_13_	A_6_	A_13_	A_11_	A_7_	A_11_	A_7_	A_13_	A_11_	A_7_	A_13_
Nepal	A_9_	A_1_	A_11_	A_9_	A_11_	A_13_	A_11_	A_9_	A_13_	A_11_	A_13_	A_10_	A_11_	A_9_	A_13_
Pakistan	A_7_	A_6_	A_9_	A_9_	A_7_	A_6_	A_9_	A_6_	A_7_	A_9_	A_6_	A_7_	A_9_	A_6_	A_7_
India	A_9_	A_6_	A_5_	A_9_	A_6_	A_5_	A_9_	A_6_	A_5_	A_9_	A_6_	A_5_	A_9_	A_6_	A_10_
Bangladesh	A_9_	A_11_	A_1_	A_7_	A_11_	A_1_	A_7_	A_9_	A_1_	A_9_	A_7_	A_5_	A_7_	A_5_	A_8_
Sri Lanka	A_9_	A_7_	A_6_	A_9_	A_7_	A_5_	A_7_	A_11_	A_6_	A_11_	A_6_	A_7_	A_11_	A_9_	A_6_
Kazakhstan	A_8_	A_11_	A_3_	A_8_	A_2_	A_12_	A_8_	A_1_	A_2_	A_12_	A_2_	A_8_	A_2_	A_1_	A_6_
Kyrgyzstan	A_13_	A_6_	A_8_	A_13_	A_6_	A_3_	A_13_	A_6_	A_3_	A_13_	A_6_	A_5_	A_13_	A_6_	A_5_
Tajikistan	A_13_	A_11_	A_6_	A_11_	A_6_	A_13_	A_11_	A_6_	A_1_	A_11_	A_6_	A_5_	A_11_	A_6_	A_5_
Uzbekistan	A_1_	A_8_	A_9_	A_8_	A_1_	A_11_	A_6_	A_8_	A_11_	A_6_	A_8_	A_5_	A_6_	A_5_	A_13_
Turkmenistan	A_11_	A_8_	A_2_	A_11_	A_8_	A_2_	A_11_	A_8_	A_2_	A_11_	A_5_	A_8_	A_11_	A_5_	A_8_
Afghanistan	A_13_	A_7_	A_10_	A_13_	A_7_	A_9_	A_13_	A_9_	A_7_	A_13_	A_7_	A_9_	A_13_	A_9_	A_7_
Syria	A_11_	A_7_	A_10_	A_11_	A_7_	A_10_	A_13_	A_9_	A_10_	A_13_	A_11_	A_9_	A_13_	A_11_	A_9_
Kuwait	A_6_	A_3_	A_8_	A_3_	A_6_	A_2_	A_10_	A_3_	A_6_	A_6_	A_9_	A_3_	A_6_	A_3_	A_2_
Israel	A_13_	A_9_	A_7_	A_13_	A_10_	A_7_	A_13_	A_10_	A_5_	A_13_	A_7_	A_5_	A_13_	A_7_	A_5_
Iraq	A_13_	A_9_	A_7_	A_9_	A_7_	A_11_	A_9_	A_7_	A_11_	A_7_	A_9_	A_11_	A_9_	A_11_	A_7_
Iran	A_11_	A_6_	A_10_	A_11_	A_10_	A_6_	A_10_	A_11_	A_6_	A_11_	A_6_	A_8_	A_11_	A_10_	A_6_
Azerbaijan	A_10_	A_2_	A_1_	A_10_	A_7_	A_8_	A_10_	A_7_	A_11_	A_10_	A_7_	A_11_	A_10_	A_7_	A_11_
Oman	A_11_	A_6_	A_3_	A_11_	A_3_	A_6_	A_3_	A_11_	A_6_	A_11_	A_3_	A_2_	A_11_	A_12_	A_2_
Lebanon	A_7_	A_10_	A_6_	A_7_	A_10_	A_9_	A_9_	A_10_	A_7_	A_7_	A_10_	A_8_	A_7_	A_12_	A_10_
Qatar	A_10_	A_11_	A_7_	A_10_	A_11_	A_7_	A_10_	A_11_	A_3_	A_10_	A_11_	A_7_	A_10_	A_12_	A_11_
Armenia	A_10_	A_7_	A_11_	A_10_	A_13_	A_7_	A_10_	A_11_	A_7_	A_10_	A_13_	A_11_	A_13_	A_10_	A_11_
Turkey	A_9_	A_5_	A_10_	A_7_	A_10_	A_9_	A_10_	A_7_	A_9_	A_9_	A_7_	A_1_	A_7_	A_9_	A_5_
Jordan	A_9_	A_3_	A_13_	A_3_	A_4_	A_13_	A_13_	A_3_	A_4_	A_13_	A_4_	A_9_	A_13_	A_7_	A_4_
Yemen	A_8_	A_1_	A_9_	A_1_	A_9_	A_8_	A_9_	A_1_	A_8_	A_13_	A_9_	A_1_	A_1_	A_10_	A_9_
Saudi Arabia	A_9_	A_6_	A_8_	A_8_	A_6_	A_1_	A_8_	A_6_	A_10_	A_9_	A_10_	A_8_	A_10_	A_9_	A_1_
China	A_9_	A_11_	A_1_	A_9_	A_7_	A_11_	A_9_	A_7_	A_10_	A_10_	A_9_	A_7_	A_7_	A_11_	A_10_
Mongolia	A_11_	A_13_	A_3_	A_13_	A_11_	A_3_	A_11_	A_12_	A_13_	A_12_	A_11_	A_13_	A_12_	A_3_	A_11_
Russia	A_9_	A_11_	A_1_	A_9_	A_10_	A_11_	A_9_	A_11_	A_8_	A_11_	A_10_	A_8_	A_11_	A_10_	A_8_
Italy	A_4_	A_9_	A_12_	A_4_	A_12_	A_2_	A_12_	A_4_	A_2_	A_12_	A_4_	A_2_	A_9_	A_12_	A_4_
Netherlands	A_12_	A_6_	A_2_	A_12_	A_3_	A_6_	A_12_	A_2_	A_3_	A_12_	A_3_	A_2_	A_12_	A_3_	A_4_
Austria	A_12_	A_11_	A_2_	A_12_	A_11_	A_4_	A_11_	A_12_	A_4_	A_11_	A_12_	A_3_	A_11_	A_12_	A_9_
Belgium	A_12_	A_3_	A_5_	A_12_	A_3_	A_5_	A_12_	A_3_	A_5_	A_12_	A_3_	A_9_	A_12_	A_3_	A_5_
France	A_12_	A_9_	A_5_	A_12_	A_5_	A_9_	A_12_	A_5_	A_9_	A_12_	A_9_	A_5_	A_12_	A_9_	A_5_
Spain	A_9_	A_12_	A_4_	A_12_	A_9_	A_4_	A_12_	A_4_	A_2_	A_12_	A_4_	A_2_	A_12_	A_4_	A_9_
Portugal	A_12_	A_4_	A_10_	A_12_	A_4_	A_10_	A_12_	A_4_	A_10_	A_12_	A_4_	A_10_	A_12_	A_4_	A_10_
United Kingdom	A_12_	A_10_	A_9_	A_12_	A_10_	A_9_	A_12_	A_9_	A_10_	A_12_	A_9_	A_2_	A_12_	A_9_	A_4_
Ireland	A_12_	A_3_	A_2_	A_12_	A_3_	A_2_	A_12_	A_3_	A_2_	A_12_	A_3_	A_2_	A_12_	A_3_	A_4_
Switzerland	A_12_	A_4_	A_3_	A_12_	A_3_	A_4_	A_12_	A_3_	A_4_	A_12_	A_2_	A_3_	A_12_	A_3_	A_4_
Greece	A_12_	A_4_	A_9_	A_9_	A_12_	A_4_	A_12_	A_9_	A_4_	A_12_	A_9_	A_4_	A_12_	A_9_	A_4_
Germany	A_12_	A_4_	A_5_	A_12_	A_4_	A_5_	A_12_	A_4_	A_2_	A_12_	A_9_	A_4_	A_9_	A_12_	A_4_
Poland	A_4_	A_10_	A_2_	A_4_	A_2_	A_3_	A_4_	A_3_	A_2_	A_3_	A_4_	A_2_	A_3_	A_4_	A_2_
Croatia	A_4_	A_10_	A_2_	A_4_	A_12_	A_2_	A_12_	A_4_	A_2_	A_3_	A_4_	A_2_	A_3_	A_4_	A_2_
Czech Republic	A_3_	A_5_	A_4_	A_3_	A_4_	A_2_	A_3_	A_4_	A_2_	A_3_	A_4_	A_2_	A_3_	A_4_	A_2_
Slovakia	A_3_	A_5_	A_4_	A_3_	A_5_	A_4_	A_3_	A_5_	A_4_	A_3_	A_5_	A_4_	A_3_	A_4_	A_5_
Albania	A_1_	A_4_	A_13_	A_13_	A_2_	A_4_	A_2_	A_13_	A_4_	A_2_	A_4_	A_13_	A_2_	A_4_	A_8_
Bulgaria	A_10_	A_2_	A_4_	A_10_	A_12_	A_3_	A_3_	A_10_	A_4_	A_3_	A_10_	A_4_	A_3_	A_4_	A_2_
Hungary	A_3_	A_2_	A_12_	A_3_	A_12_	A_4_	A_3_	A_12_	A_4_	A_2_	A_3_	A_12_	A_2_	A_3_	A_12_
Lithuania	A_3_	A_4_	A_2_	A_3_	A_4_	A_5_	A_3_	A_4_	A_2_	A_3_	A_4_	A_2_	A_3_	A_4_	A_2_
Ukraine	A_11_	A_10_	A_4_	A_11_	A_10_	A_4_	A_11_	A_1_	A_4_	A_11_	A_10_	A_9_	A_11_	A_10_	A_1_
Moldova	A_5_	A_11_	A_3_	A_11_	A_5_	A_8_	A_11_	A_5_	A_13_	A_11_	A_5_	A_13_	A_11_	A_5_	A_13_
Romania	A_5_	A_4_	A_2_	A_5_	A_4_	A_2_	A_5_	A_4_	A_2_	A_5_	A_4_	A_3_	A_5_	A_4_	A_3_
Slovenia	A_3_	A_4_	A_5_	A_3_	A_12_	A_5_	A_3_	A_12_	A_5_	A_3_	A_5_	A_4_	A_3_	A_5_	A_4_
Belarus	A_11_	A_8_	A_7_	A_11_	A_7_	A_10_	A_11_	A_7_	A_10_	A_11_	A_7_	A_3_	A_11_	A_7_	A_3_
Serbia	A_11_	A_5_	A_1_	A_11_	A_5_	A_8_	A_11_	A_5_	A_8_	A_11_	A_5_	A_3_	A_11_	A_5_	A_3_
Egypt	A_9_	A_7_	A_6_	A_7_	A_6_	A_9_	A_7_	A_9_	A_6_	A_7_	A_9_	A_6_	A_7_	A_9_	A_6_
Algeria	A_9_	A_6_	A_7_	A_9_	A_6_	A_7_	A_9_	A_6_	A_7_	A_7_	A_6_	A_8_	A_7_	A_6_	A_9_
Morocco	A_9_	A_11_	A_4_	A_11_	A_4_	A_7_	A_11_	A_4_	A_8_	A_11_	A_4_	A_13_	A_11_	A_4_	A_7_
Tunisia	A_11_	A_8_	A_7_	A_11_	A_8_	A_3_	A_11_	A_1_	A_8_	A_11_	A_8_	A_1_	A_11_	A_8_	A_9_
Libya	A_11_	A_7_	A_8_	A_11_	A_7_	A_3_	A_9_	A_11_	A_1_	A_9_	A_1_	A_11_	A_1_	A_11_	A_7_
Sudan	A_7_	A_11_	A_6_	A_7_	A_11_	A_9_	A_7_	A_11_	A_10_	A_7_	A_11_	A_10_	A_13_	A_7_	A_11_
Ethiopia	A_13_	A_7_	A_10_	A_13_	A_7_	A_10_	A_13_	A_7_	A_10_	A_7_	A_13_	A_11_	A_7_	A_11_	A_1_
Kenya	A_11_	A_9_	A_1_	A_11_	A_9_	A_1_	A_9_	A_13_	A_11_	A_11_	A_9_	A_8_	A_11_	A_9_	A_13_
Tanzania	A_13_	A_11_	A_6_	A_13_	A_11_	A_6_	A_13_	A_11_	A_6_	A_11_	A_13_	A_6_	A_11_	A_9_	A_6_
New Zealand	A_5_	A_4_	A_2_	A_5_	A_4_	A_2_	A_5_	A_4_	A_2_	A_5_	A_4_	A_2_	A_9_	A_5_	A_4_
Australia	A_5_	A_12_	A_10_	A_12_	A_2_	A_5_	A_12_	A_2_	A_5_	A_12_	A_2_	A_5_	A_12_	A_5_	A_9_

## Data Availability

Not applicable.

## References

[1] Liu W.D., Dunford M., Gao B.Y. (2018). A discursive construction of the Belt and Road Initiative: From neo-liberal to inclusive globalization. J. Geogr. Sci..

[2] Jacob J.T. (2017). China’s Belt and Road Initiative: Perspectives from India. China. World. Econ..

[3] Rolland N. (2017). China’s "Belt and Road Initiative": Underwhelming or game-changer?. Wash. Q..

[4] Nanwani S. (2019). Belt and Road Initiative: Responses from Japan and India—Bilateralism, Multilateralism and Collaborations. Glob. Policy.

[5] Blanchard J.M.F., Flint C. (2017). The geopolitics of China’s Maritime Silk Road Initiative. Geopolitics.

[6] Shen L.H., Hong Y.R. (2023). Can geopolitical risks excite Germany economic policy uncertainty: Rethinking in the context of the Russia-Ukraine conflict. Financ. Res. Lett..

[7] Shahzad U., Ramzan M., Shah M.I., Doğan B., Ajmi A.N. (2022). Analyzing the Nexus Between Geopolitical Risk, Policy Uncertainty, and Tourist Arrivals: Evidence from the United States. Eval. Rev..

[8] Suder G. (2006). Corporate Strategies under International Terrorism and Adversity.

[9] Zhang Z., He M., Zhang Y., Wang Y. (2022). Geopolitical risk trends and crude oil price predictability. Energy.

[10] Zhao W., Zhong R., Sohail S., Majeed M.T., Ullah S. (2021). Geopolitical risks, energy consumption, and CO_2_ emissions in BRICS: An asymmetric analysis. Environ. Sci. Pollut. Res..

[11] Jiang Y.H., Tian G.Y., Wu Y.Q., Mo B. (2022). Impacts of geopolitical risks and economic policy uncertainty on Chinese tourism-isted company stock. Int. J. Financ. Econ..

[12] Kiik L. (2016). Nationalism and anti-ethno-politics: Why ‘Chinese development’ failed at Myanmar’s Myitsone dam. Eurasian Geogr. Econ..

[13] Cheng C.H.J., Chiu C.-W. (2018). How important are global geopolitical risks to emerging countries?. Int. Econ..

[14] Caniglia L. (2011). Western ostracism and China’s presence in Africa. China Inf..

[15] Shmueli D.F., Collins K.N., Ben G.M. (2014). Conflict over sacred space: The case of Nazareth. Cities.

[16] Yang M., Zhang Q., Yi A., Peng P. (2021). Geopolitical risk and stock market volatility in emerging economies: Evidence from GARCH-MIDAS model. Discrete. Dyn. Nat. Soc..

[17] Meehan K., Klenk N.L., Mendez F. (2018). The geopolitics of climate knowledge mobilization: Transdisciplinary research at the science–policy interface(s) in the Americas. Science.

[18] Kim K. (2021). Changes in US security and defense strategy toward China: Assessment and policy implications. Korean J. Def. Anal..

[19] Hu W., Ge Y., Hu Z., Ye S., Yang F., Jiang H., Hou K., Deng Y. (2022). Geo-Economic Linkages between China and the Countries along the 21st-Century Maritime Silk Road and Their Types. Int. J. Environ. Res. Public. Health..

[20] Mamun A., Uddin G.S., Suleman M.T., Kang S.H. (2019). Geopolitical risk, uncertainty and Bitcoin investment. Phys. A Stat. Mech. Its Appl..

[21] Zhang Y., Hamori S. (2022). A connectedness analysis among BRICS’s geopolitical risks and the US macroeconomy. Econ. Anal. Policy.

[22] Saâdaoui F., Ben Jabeur S., Goodell J.W. (2022). Causality of geopolitical risk on food prices: Considering the Russo–Ukrainian conflict. Financ. Res. Lett..

[23] Zhang Z., Bouri E., Klein T., Jalkh N. (2022). Geopolitical risk and the returns and volatility of global defense companies: A new race to arms?. Int. Rev. Financ. Anal..

[24] Le A.-T., Tran T.P. (2021). Does geopolitical risk matter for corporate investment? Evidence from emerging countries in Asia. J. Multinatl. Financ. Manag..

[25] Cepni O., Emirmahmutoglu F., Guney I.E., Yilmaz M.H. (2022). Do the carry trades respond to geopolitical risks? Evidence from BRICS countries. Econ. Syst..

[26] Wu S., Zhang Y., Yan J. (2022). Comprehensive assessment of geopolitical risk in the Himalayan region based on the grid scale. Sustainability.

[27] Jiyoun A.N., Hyunwoo R.O.H. (2018). Measuring North Korean geopolitical risks: Implications for Asia’s financial volatilities. East Asian Policy.

[28] Dziedzic A., Riad A., Tanasiewicz M., Attia S. (2022). The increasing population movements in the 21st Century: A call for the E-register of health-related data integrating health care systems in Europe. Int. J. Environ. Res. Public Health.

[29] Costola M., Donadelli M., Gerotto L., Gufler I. (2022). Global risks, the macroeconomy, and asset prices. Empir. Econ..

[30] Faruk B., Hatice O.B., Mudassar H., Russell G.A. (2022). Geopolitical risk spillovers and its determinants. Ann. Reg. Sci..

[31] The Economist Intelligence Unit (2015). Prospects and Challenges on China’s "One Belt, One Road": A Risk Assessment Report.

[32] Marsh (2018). Political Risk Map 2018: Tensions and Turbulence Ahead.

[33] Zhou Q., He Z., Yang Y. (2020). Energy geopolitics in Central Asia: China’s involvement and responses. J. Geogr. Sci..

[34] Zhang X. (2017). Chinese Capitalism and the Maritime Silk Road: A world-systems perspective. Geopolitics.

[35] Kong L.J. (2015). The Belt and Road Initiative and China’s foreign policy toward its territorial and boundary disputes. China. Q. Int. Strateg. Stud..

[36] Ataka H. (2016). Geopolitics or Geobody Politics? Understanding the Rise of China and Its Actions in the South China Sea. Asian J. Peacebuild..

[37] Zhang C., Xiao C., Liu H. (2019). Spatial big data analysis of political risks along the Belt and Road. Sustainability.

[38] Yu Z., Xiao Y., Li J.P. (2021). How does geopolitical uncertainty affect Chinese overseas investment in the energy sector? Evidence from the South China Sea Dispute. Energy Econ..

[39] Weng L.F., Xue L., Sayer J., Riggs R.A., Langston J.D., Boedhihartono A.K. (2021). Challenges faced by Chinese firms implementing the ‘Belt and Road Initiative’: Evidence from three railway project. Res. Glob..

[40] Flint C., Zhu C.P. (2019). The geopolitics of connectivity, cooperation, and hegemonic competition: The Belt and Road Initiative. Geoforum.

[41] Gill S. (1988). The rise and decline of great powers: The American case. Politics.

[42] Dijkink G. (2006). When geopolitics and religion fuse: A historical perspective. Geopolitics.

[43] Jiang W.L., Martek I. (2021). Political risk analysis of foreign direct investment into the energy sector of developing countries. J. Clean. Prod..

[44] Liu J., Ma F., Tang Y., Zhang Y. (2019). Geopolitical risk and oil volatility: A new insight. Energy Econ..

[45] Coleman M. (2003). The naming of ‘terrorism’ and evil ‘outlaws’: Geopolitical place-making after 11 September. Geopolitics.

[46] Liu Z., Jiang Z., Xu C., Cai G., Zhan J. (2021). Assessment of provincial waterlogging risk based on entropy weight TOPSIS–PCA method. Nat. Hazards.

[47] Ou Z.R., Zhu Q.K., Sun Y.Y. (2017). Regional ecological security and diagnosis of obstacle factors in underdeveloped regions: A case study in Yunnan province, China. J. Mt. Sci..

[48] Pulkkinen A., Rastätter L. (2009). Minimum variance analysis-based propagation of the solar wind observations: Application to real-time global magnetohydrodynamic simulations. Space Weather.

[49] Richardson P.B. (2021). Geopolitical encounters and entanglements along the Belt and Road Initiative. Geogr. Compass.

[50] Kamenopoulos S.N., Agioutantis Z. (2019). Geopolitical risk assessment of countries with rare earth element deposits. Min. Met. Explor..

[51] Liu H.M., Hu S.L., Fang K., He G.Q., Ma H.T., Cui X.G. (2019). A comprehensive assessment of political, economic and social risks and their prevention for the countries along the Belt and Road. Geogr. Res..

